# Why does a point of care guided transfusion algorithm not improve blood loss and transfusion practice in patients undergoing high-risk cardiac surgery? A prospective randomized controlled pilot study

**DOI:** 10.1186/s12871-019-0689-7

**Published:** 2019-02-18

**Authors:** F. Lehmann, J. Rau, B. Malcolm, M. Sander, C. von Heymann, T. Moormann, T. Geyer, F. Balzer, K. D. Wernecke, L. Kaufner

**Affiliations:** 1 Department of Anaesthesiology and Intensive Care Medicine, Charité – Universitätsmedizin Berlin, Campus Virchow-Klinikum and Campus Charité Mitte, Berlin, Germany; 2 Division of Medical Biotechnology, Paul-Ehrlich-Institut, Federal Institute for Vaccines and Biomedicines, Langen, Hessen Germany; 3 Department of Internal Medicine, Hegau-Bodensee-Klinikum, Singen, Baden-Württemberg Germany; 4 Department of Anaesthesiology, Intensive Care Medicine and Pain therapy, University Hospital Gießen UKGM, Justus-Liebig-University Giessen, Giessen, Hessen Germany; 5 Department of Anaesthesia, Intensive Care Medicine, Emergency Medicine and Pain Therapy, Vivantes Klinikum im Friedrichshain, Berlin, Germany; 6 Department of Anaesthesiology and Intensive Care Medicine, Martin-Luther-Krankenhaus, Berlin, Germany; 7 CRO SOSTANA GmbH and Charité – Universitätsmedizin Berlin, Berlin, Germany

**Keywords:** Algorithms, Blood coagulation, Blood transfusion, Point-of-care systems, Thoracic surgery

## Abstract

**Background:**

Adult cardiac surgery is often complicated by elevated blood losses that account for elevated transfusion requirements. Perioperative bleeding and transfusion of blood products are major risk factors for morbidity and mortality. Timely diagnostic and goal-directed therapies aim at the reduction of bleeding and need for allogeneic transfusions.

**Methods:**

Single-centre, prospective, randomized trial assessing blood loss and transfusion requirements of 26 adult patients undergoing elective cardiac surgery at high risk for perioperative bleeding. Primary endpoint was blood loss at 24 h postoperatively. Random assignment to intra- and postoperative haemostatic management following either an algorithm based on conventional coagulation assays (conventional group: platelet count, aPTT, PT, fibrinogen) or based on point-of-care (PoC-group) monitoring, i.e. activated rotational thromboelastometry (ROTEM®) combined with multiple aggregometry (Multiplate®). Differences between groups were analysed using nonparametric tests for independent samples.

**Results:**

The study was terminated after interim analysis (*n* = 26). Chest tube drainage volume was 360 ml (IQR 229-599 ml) in the conventional group, and 380 ml (IQR 310-590 ml) in the PoC-group (*p* = 0.767) after 24 h. Basic patient characteristics, results of PoC coagulation assays, and transfusion requirements of red blood cells and fresh frozen plasma did not differ between groups. Coagulation results were comparable. Platelets were transfused in the PoC group only.

**Conclusion:**

Blood loss via chest tube drainage and transfusion amounts were not different comparing PoC- and central lab-driven transfusion algorithms in subjects that underwent high-risk cardiac surgery. Routine PoC coagulation diagnostics do not seem to be beneficial when actual blood loss is low. High risk procedures might not suffice as a sole risk factor for increased blood loss.

**Trial registration:**

NCT01402739, Date of registration July 26, 2011.

## Background

Adult cardiac surgery patients are at increased risk of perioperative bleeding, either due to concomitant medication [1, 2] or the type of surgery itself [3]. Increased blood loss is associated with increased need for allogeneic blood transfusions [4] and poorer outcome [5, 6]. Minimising blood loss and bleeding is the second pillar of patient blood management [7, 8], a multimodal concept that is encouraged by the World Health Organization [9]. Haemostasis management is one aspect to minimize blood loss [7]. For red blood cells, algorithm-based transfusion is considered safe and associated with comparably low transfusion rates [6, 10, 11].

Enforceable transfusion algorithms are part of the recommendation on the management of blood resources of the 2011 Update to The Society of Thoracic Surgeons and the Society of Cardiovascular Anesthesiologists Blood Conservation Clinical Practice Guidelines [12], and the recent guideline of the European Association of Cardiothoracic Anaesthesiology (EACTA) in conjunction with the European Association of Cardio-thoracic Surgeons (EACTS) [13]. The use of transfusion algorithms itself has been shown to significantly reduce patients’ exposure to blood products in cardiac surgery [13–16].

Perioperative monitoring of coagulation may help to identify the underlying causes for bleeding and enable specific treatment. Point-of-care (PoC) coagulation monitoring is widely used and has been recommended in various guidelines [12, 13, 17–19].

As reviewed earlier, several studies focused on PoC-guided transfusion algorithms compared to algorithms based on conventional coagulation testing, clinician discretion or “standard of care” in cardiac surgery [14, 20]. Few were randomized controlled trials [15, 21–26]. Only two studies applied explicit transfusion algorithms in both, the PoC- and conventional coagulation tests-guided group [23, 24]. Of those, one study applied the transfusion protocols inside the operation theatre only [23], although a relevant number of bleeding episodes and transfusions occurs postoperatively [6, 24].

Any type of algorithm allows defining a goal-directed transfusion strategy [16]. Furthermore, the implementation of an algorithm itself has been shown to improve standardisation and outcome [27]. The question remains, whether the superior results of PoC-guided transfusion algorithms [20] are reproducible in a setting of restrictive and explicit transfusion algorithms both in PoC- and conventional coagulation assay-guided protocols.

Therefore, we conducted a prospective randomized trial in cardiac surgical procedures at high risk for bleeding that compared transfusion algorithms either guided by PoC (activated rotational thromboelastometry, and multiple electrode aggregometry (MEA)) or standard coagulation assays. We assumed that PoC coagulation monitoring is superior to standard coagulation measurements due to shorter turnaround times of test results enabling a faster and more specific treatment with haemostatic products. Our hypothesis was a reduction of postoperative blood loss via chest tube drainage and transfusion needs in patients undergoing high-risk cardiac surgery treated following a PoC-driven transfusion protocol compared to a central lab-guided protocol.

## Methods

This single centre trial has a prospective, randomized parallel-group design with two study arms (*n* = 100, Fig. 2). First study arm focused on patients undergoing high risk cardiac surgery (*n* = 50, Fig. 2, data shown). Second study arm investigated patients with dual platelet inhibition undergoing cardiac surgery (n = 50, Fig. 2, data not shown). The study was conducted at the Department of Anaesthesiology and Intensive Care Medicine of Charité - University Medicine Berlin, Berlin, Germany in adherence to the latest version of the declaration of Helsinki, approved by the local Ethics Board (Ethics Committee of Charité - University Medicine Berlin (EA1/263/10). The trial is registered with clinicaltrials.gov (identifier NCT01402739) and adheres to CONSORT guidelines. All patients gave written informed consent prior to entering the study.

For the first study arm, patients were eligible for participation if aged 18-80 yrs. and scheduled for cardiac surgery using cardio-pulmonary bypass (CPB) for either a combined CABG/valve procedure, a double or triple valve procedure or a redo surgery, defining patients as at high risk for bleeding and transfusion [3]. Key exclusion criteria were hereditary or acquired defects in haemostasis (see Appendix for all exclusion criteria) and surgical procedures not regarded as of high risk of bleeding. After enrolment, participants were assigned to the PoC or the conventional group by simple, stratified envelope randomization with allocation concealment aiming at 1:1 assignment. The stratum was risk for bleeding, and the person conducting randomization was neither the person enrolling the patients, nor one of the treating physicians. The primary outcome parameter was the chest tube drainage volume after 24 h. Secondary outcome parameters included the course of chest tube drainage at 6, 12 and 24 h postoperatively, the need of allogeneic blood transfusions in the first 24 h, the course of conventional coagulation parameters, the duration of mechanical ventilation, and the incidence of renal replacement therapy.

### Perioperative management

Anaesthetic, surgical, CPB and postoperative intensive care management were standardised for each patient. Changes to local standard management applied to coagulation monitoring and transfusion management according to the study protocol only. Intraoperative blood losses were salvaged and washed before retransfusion to avoid heparin effects.

Anaesthetic management was conducted following local standard operating procedures for cardiac surgery patients. Routine monitoring included continuous arterial blood pressure and central venous pressure monitoring, 5-lead ECG, pulse-oximetry and BIS monitoring. General anaesthesia was induced with etomidate 0.2–0.3 mg/kg body weight (kgBW), sufentanil 0.2–0.4 μg/kgBW and cis-atracurium 0.1–0.15 mg/kgBW. Anaesthesia was maintained with sufentanil 0.5–1.0 μg/kgBW/h and sevoflurane 0.7–1 MAC. During normothermic CPB in addition to sufentanil, propofol 2-4 mg/kgBW/h was infused to ensure sufficient anaesthetic depth. Tranexamic acid was dosed according to the BART protocol in all patients [28].

Blood samples for coagulation monitoring were taken from the arterial line prior to induction, after the start of CPB, after aortic declamping and administration of protamine as well as at 1 h, 6 h, 24 h and 48 h postoperatively.

### Transfusion protocols

Transfusion protocols were used from induction of general anaesthesia until 24 h postoperatively:

- *Packed red blood cells (RBC)*. For both groups, a previously described protocol used safely in moderate-risk patients undergoing cardiac surgery was applied [10]. Briefly, a haemoglobin of ≤6 g/dl led to a transfusion of one unit RBC. For haemoglobin values 6-8 g/dl, transfusion was acceptable, but not mandatory. For haemoglobin values of 8-10 g/dl, transfusion of one unit RBC was acceptable if at least one of the following was present: ScvO2 < 70% if arterial SpO2 > 90%, cardiac index < 2.5 refractory towards inotropes or mechanical support, or symptoms of end-organ ischemia.
- *Fresh frozen plasma (FFP), platelet concentrates, and fibrinogen concentrate*. For both groups, transfusion of these blood products was allowed either before chest closure in case of severe diffuse bleeding delaying chest closure as assessed by the cardiac surgeon and anaesthesiologist, or in case of blood loss exceeding 1.5 ml/kgBW/hour in two consecutive hours or 4 ml/kgBW/hour for at least 30 min postoperatively.

In the conventional group, 10 ml/kgBW FFP was transfused if INR > 1.5. A platelet count of ≤100/nl led to a transfusion of one apheresis platelet concentrate unit. If fibrinogen was ≤150 mg/dl, 2 g of fibrinogen concentrate were administered. Twenty-five mg of Protamine could be administered in patients whose ACT was > 10% or whose aPTT was > 20% above the upper reference range (120 s. and 26–40 s., respectively). In cases of persistent bleeding despite surgical haemostasis or after prolonged CPB time, or pre-operative antiplatelet medication, platelets and/or FFP were acceptable at the discretion of the attending anaesthesiologist. In cases of normal conventional coagulation parameters and a suspected platelet disorder desmopressin 0.3 μg/kgBW was considered.

**Fig. 1:**
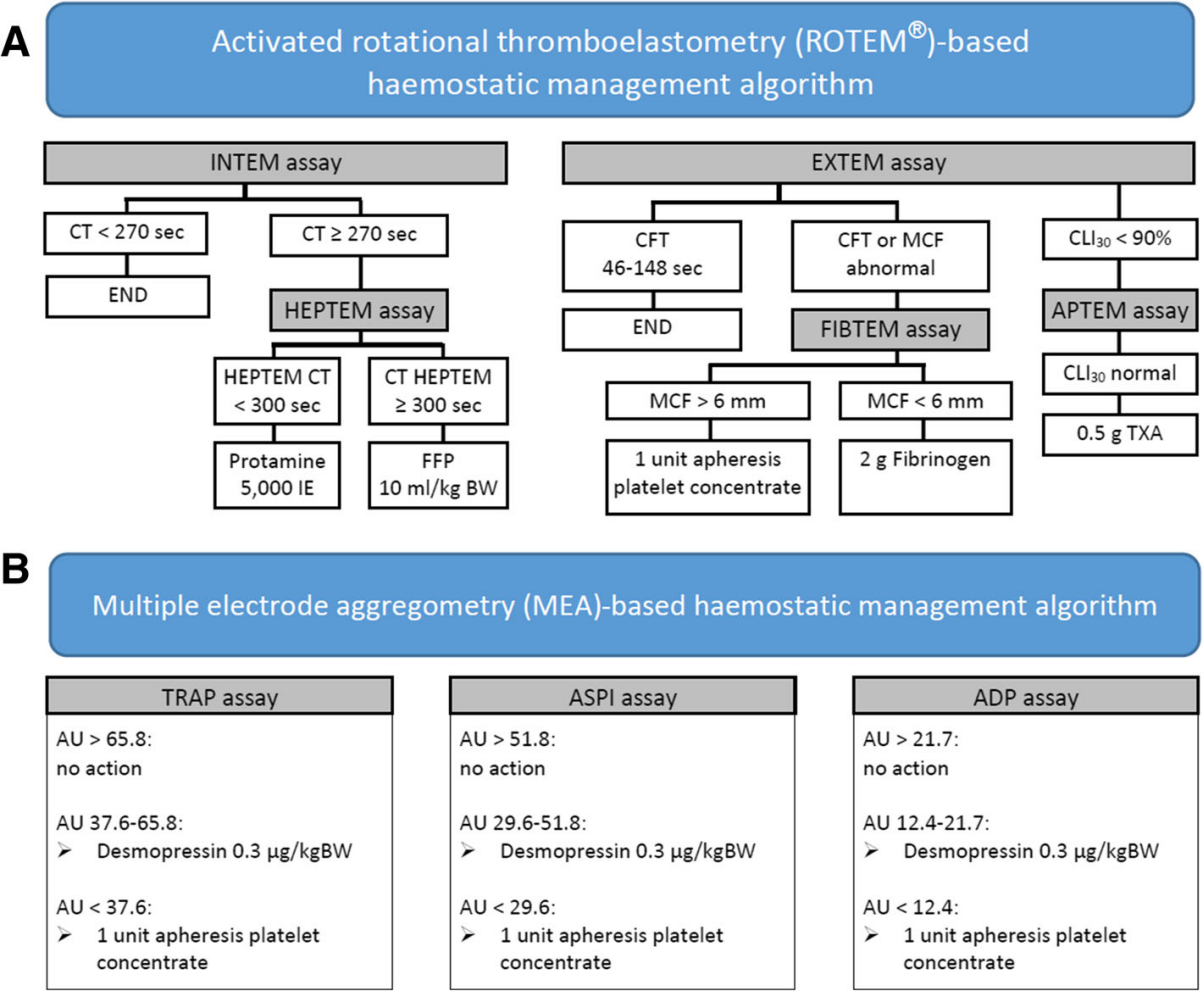
PoC therapy algorithms for the PoC group in case of blood loss exceeding 1.5 ml/kg body weight (kgBW)/hour in two consecutive hours or 4 ml/kgBW/hour for at least 30 min, or bleeding delaying chest closure intraoperatively. **a** Activated rotational thromboelastometry (ROTEM®)-based haemostatic management algorithm. **b** Multiple electrode aggregometry (MEA)-based haemostatic management algorithm

In the PoC group, parallel measurements using the activated rotational thromboelastometry (ROTEM™, TEM International GmbH, Munich, Germany) and MEA (Multiplate® Analyzer, Roche Deutschland, Mannheim, Germany) were performed. Activated rotational thromboelastometry measures clot-building, clot firmness, clot lysis and their dynamics in recalcified citrated whole blood [29]. MEA reflects platelet aggregation ability in anticoagulated whole blood, being able to differentiate platelet inhibition by acetylsalicylic acid, thienopyridines and GPIIbIIIa inhibitors [30]. The haemostatic management algorithms are given in Fig. 1. Additionally, in cases of persistent bleeding despite normal PoC parameters desmopressin 0.3 μg/kgBW was considered if surgical bleeding was denied.

For both groups, the application of the prothrombin complex concentrate, antithrombin, recombinant factor VIIa, factors VIII, IX and XIII was acceptable in case of diagnosed deficiencies or therapy-refractory bleeding.

### Coagulation testing

In both groups, blood samples for conventional coagulation assays and point-of-care assays were collected and analysed simultaneously. Results of PoC testing were available to the attending physicians in the PoC group only but recorded for study purposes for both groups. Results of the conventional coagulation tests were automatically displayed in the electronic patient chart but were irrelevant for PoC group patients both due to later availability caused by longer turnaround times and a transfusion algorithm independent of conventional coagulation tests.

### Statistics

**Fig. 2:**
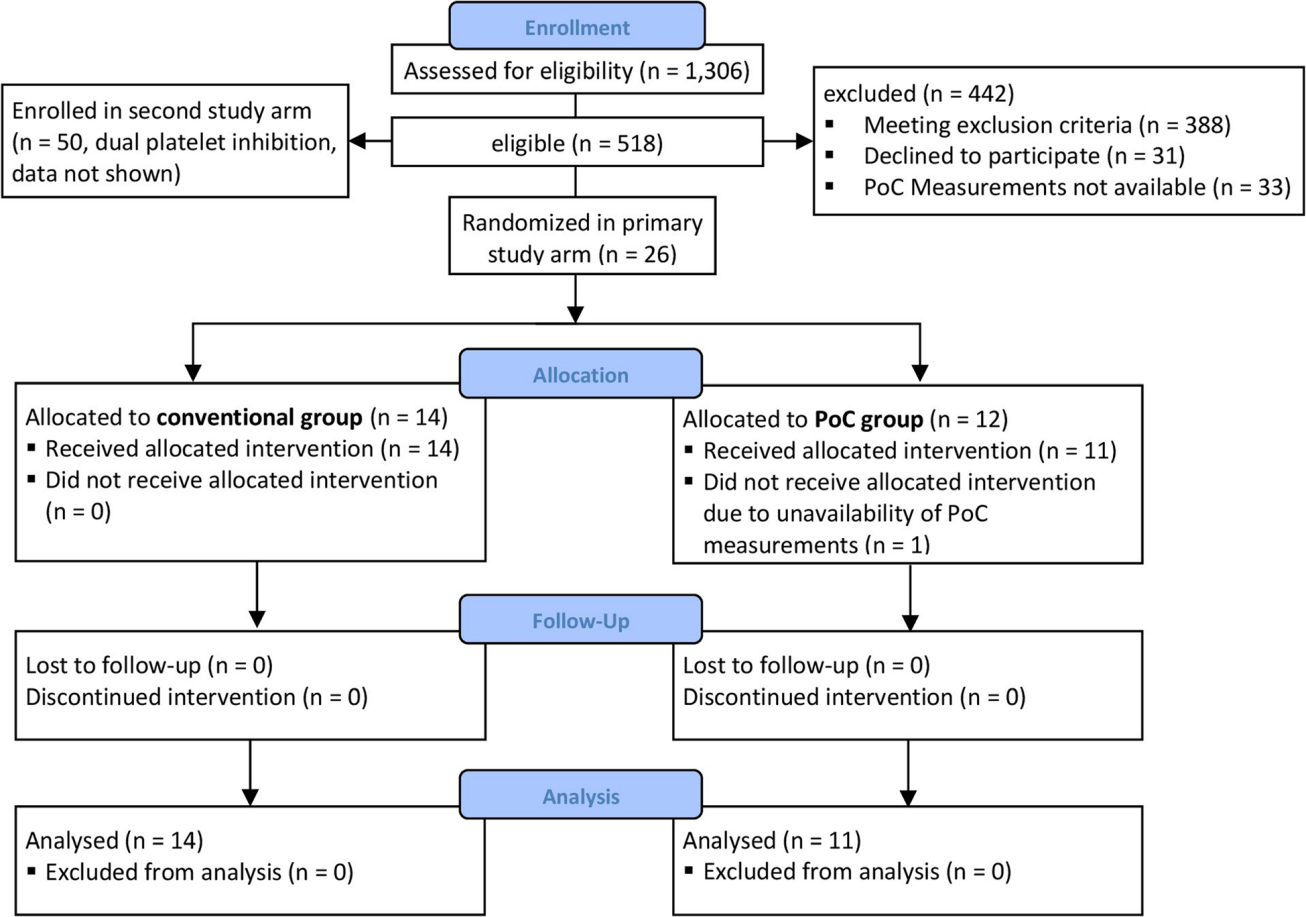
Consort flow-chart

For the first study arm (Fig. 2) we hypothesized that 250 ml difference in chest tube drainage volume would be clinically relevant. The sample size calculation performed by CRO SOSTANA GmbH, Berlin, Germany, yielded *n* = 24 per group to detect this difference over 24 h with a type one error of α = 5% (two-sided) and a power of 80%. The calculation was based on the chest tube drainage volume in the control group of a published study for a common standard deviation of 281.69 ml, investigating different cardiac surgical procedures resembling rather a high risk of bleeding population than patients undergoing one specific surgical procedure [30]. Therefore, a group size of *n* = 25 was chosen as appropriate.

An interim analysis of the primary outcome parameter blood loss in the first study arm was performed by CRO SOSTANA GmbH, Berlin, Germany, after 50% of the planned number of patients were enrolled in each group just for the purpose to validate the sample size calculation of the study arm, but not for statistical testing.

Results are given as median (interquartile ranges (IQR)). Differences between groups were analysed using the nonparametric Mann-Whitney U test or Chi-square test (categorical data) for two independent samples. Multivariate nonparametric analysis of longitudinal data in a two-factorial design (1st factor: groups, 2nd factor: repetitions in time) was used to analyse time courses. A two-tailed *p*-value < 0.05 was considered significant. Tests for secondary outcome parameters were conducted as exploratory data analysis. Therefore, no adjustments for multiple testing were applied. Calculations were performed with IBM SPSS Statistics for Windows, Versions 22.0 and 23.00, Armonk, NY: IBM Corp., or The R Project for Statistical Computing, Version 3.0.2 (2013-09-25). Propensity score matching was based on genetic matching algorithm with automated balance optimization [31] and applied with the R package “Matching” [32].

## Results

Enrolment of both study arms started August 23rd 2011. In total, 1306 patients were assessed for eligibility until March 20th 2014. Twenty-six patients were enrolled and randomized in the first arm that is described here only (results of second study arm will be described elsewhere). One patient had to be excluded pre-operatively due to technical reasons. Fourteen patients in the conventional group and 11 patients in the PoC group were analysed (Fig. 2). The first study arm was terminated early after the planned interim analysis. The revised sample size calculation for the first study arm based on the chest tube drainage volume in our patient population yielded 13,131 patients had to be recruited per group. This was considered not feasible in a single centre study.

**Fig. 3:**
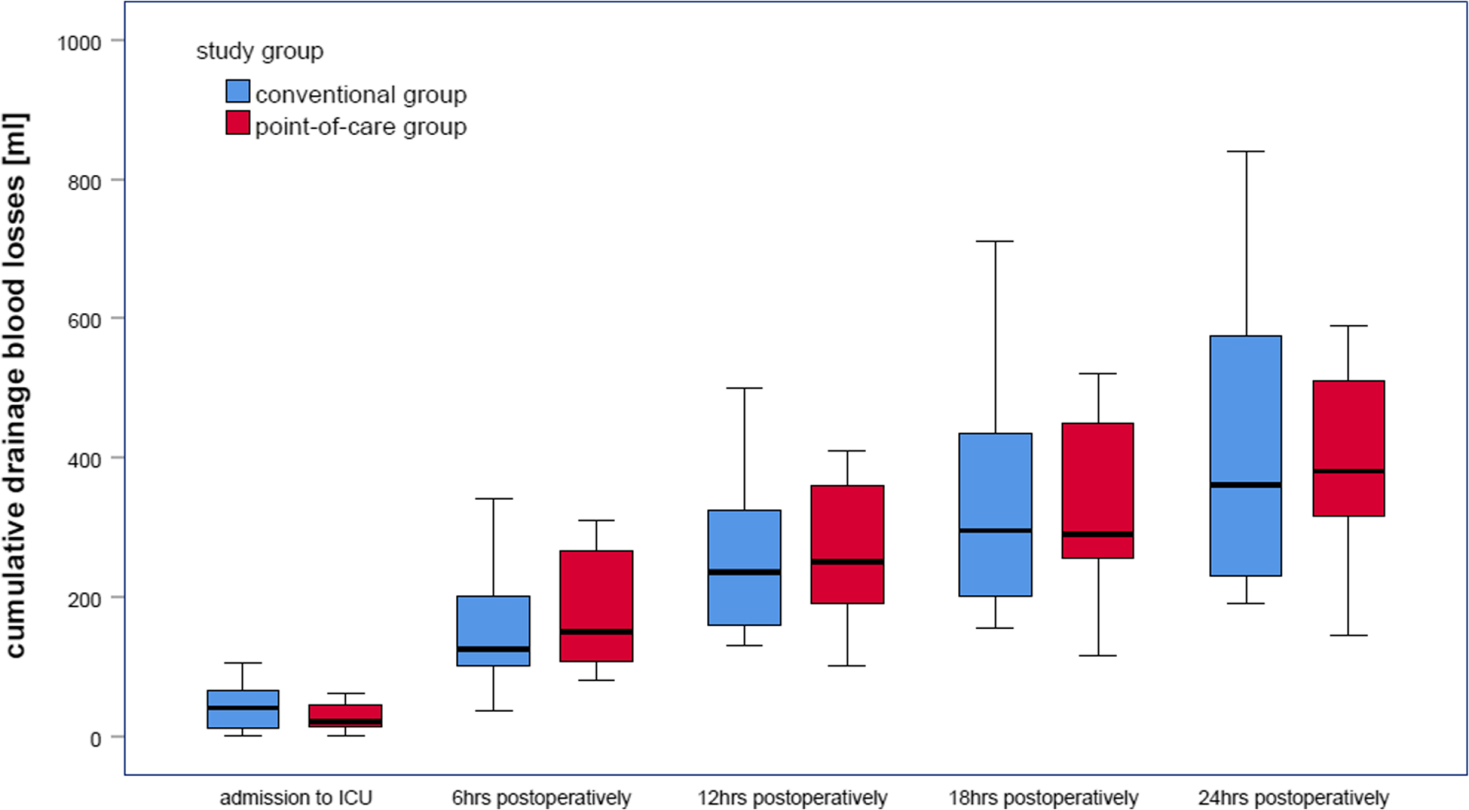
Cumulative chest tube drainage volume of the first 24 h postoperatively. Multivariate nonparametric analysis of longitudinal data in a two-factorial design (1st factor: groups, 2nd factor: repetitions in time) revealed no differences between conventional and point-of-care group (*p* = 0.548)

The basic patient and surgery characteristics are given in Table 1. There were no significant differences between groups. The primary endpoint, chest tube drainage volume at 24 h postoperatively, did not differ (*p* = 0.767). Chest tube drainage volume of the first 24 h postoperatively is given in Fig. 3, revealing no differences between groups over the time. Transfusion rates are given in Table 2, revealing transfusions of platelets in the PoC group only. The median number of platelet concentrates transfused was 2 (IQR 1.5, 2.5). Results of coagulation monitoring are given in Table 3. The only differences were a higher aPTT in the PoC group at 24 h postoperatively (*p* = 0.044), and a lower fibrinogen level in the PoC group at 6 h postoperatively (*p* = 0.006). Data of time point 6 h postoperatively is not included in Table 3.

**Table 1:** Basic patient and surgery characteristics

	Conventional group (*n* = 14)	PoC group (*n* = 11)	*P*
Sex [male]	11 (79)	6 (55)	0.397
Age [years]	70.5 (65.5–74)	71 (69–76)	0.501
BSA [m^2^]	2.07(1.98–2,12)	2.00 (1.91–2.07)	0.183
EuroScore	5 (4.5–6.3)	6 (6–8)	0.166
EF [%]	60 (54–62)	57 (50–73)	0.893
Combined CABG and valve surgery	10 (71)	7 (64)	1.000
Redo surgery	4 (29)	3 (27)	1.000
Double valve surgery	0 (0)	1 (9)	0.902
CPB time [min]	107 (87–129)	115 (99–135)	0.291
Clamping time [min]	80 (59–95)	98 (69–120)	0.344
Length of surgery [min]	209 (193–253)	225 (177–260)	0.979
Length of surgical haemostasis [min]	50 (46–61)	40 (35–52)	0.166
APACHE II	14.5 (11–17.5)	16 (11–17)	0.536
SAPS II	28 (25–32.25)	31 (25–35)	0.403
Mechanical ventilation [hours]	9 (6–16)	9 (7–12)	0.979
Incidents of RRT	0	1 (9.1)	0.902

Results are given as n (%) or median (IQR). Differences were analysed using nonparametric Mann-Whitney U for or Chi-square test two independent samples with α = 0.05 (two-sided)

*Abbreviations*: *BSA* Body surface area, *EF* Ejection fraction, *CABG* Coronary artery bypass grafting, *CPB* Cardio-pulmonary bypass, *RRT* Renal replacement therapy

**Table 2:** Total transfusion rates or amounts of salvaged blood, RBCs, FFPs, platelets, fibrinogen, PPSB, and other haemostatic agents

	Conventional group (*n* = 14)	PoC group (*n* = 11)	*P*
Retransfused, salvaged washed erythrocytes [ml]	360 (323–513)	380 (350–450)	0.936
Total number of patients transfused with RBCs	6 (43%)	8 (72%)	0.277
Thereof while on CPB	3 (21%)	3 (27%)	
Thereof intraoperatively after CPB	1 (7%)	0	
Thereof within 24 h postoperatively	1 (7%)	4 (36%)	
Thereof within 48 h postoperatively	4 (29%)	2 (18%)	
Later than 48 h postoperatively	2 (14%)	5 (45%)	
Total number of patients transfused with platelets	0	4 (36%)	0.056
Thereof intraoperatively after CPB		3 (27%)	
Thereof within 24 h postoperatively		2 (18%)	
Thereof within 48 h postoperatively		0	
Later than 48 h postoperatively		0	
Total number of PCC given	0	0	
Total number of fibrinogen concentrate given (g)	0	0	
Total number of patients transfused with FFP	1 (7%)	0	1.000
Thereof intraoperatively after CPB	1 (7%)		
Others (desmopressin, protamine), total number of patients	0	1 (9%) (desmopressin)	

Results are given as n (percentage of patients) or median (IQR), differences were analysed using Mann-Whitney U or Chi-square test for two independent samples with α = 0.05 (two-sided)

**Table 3:** Course of coagulation parameters platelet count, aPTT, PT, fibrinogen, CT (Intem), CT (Extem), MCF (Fibtem), TRAP, ASPI, and ADP

	Conventional group (*n* = 14)	PoC group (*n* = 11)	*P*
Platelet count [/nl]			
Screening	241 (207–276)	225 (201–272)	0.647
Admission to ICU	153 (111–184)	150 (120–191)	0.893
24 h postoperatively	154 (130–176)	139 (127–167)	0.979
aPTT [s]			
Pre-operatively	33.4 (31.8–38.8)	33.9 (33.3–38.5)	0.403
Admission to ICU	35.2 (33.4–37.3)	38.1 (37.3–40.9)	0.038*
24 h postoperatively	34.6 (32.4–37.9)	38.1 (34.5–43.3)	0.044*
Thromboplastin time [%]			
Pre-operatively	98 (88–104)	96 (82–101)	0.373
Admission to ICU	57 (55–65)	60 (54–62)	0.851
24 h postoperatively	77 (65–81)	67 (58–82)	0.222
Fibrinogen [g/l]			
Pre-operatively	3.98 (3.5–4.66)	3.60 (3.37–4.83)	0.467
Admission to ICU	2.58 (2.17–3.42)	2.48 (2.09–3.07)	0.699
24 h postoperatively	3.85 (3.51–4.06)	3.74 (3.53–4.5)	0.786
CT (Intem) [s]			
Pre-operatively	152 (131–179)	164 (151–185)	0.344
Admission to ICU	188 (179–201)	195 (177–213)	0.536
24 h postoperatively	157 (143–170)	166 (148–179)	0.202
CT (Extem) [s]			
Pre-operatively	52 (48–57)	60 (51–62)	0.149
Admission to ICU	64 (56–71)	66 (59–75)	0.291
24 h postoperatively	55 (48–63)	53 (46–64)	0.767
MCF (Fibtem) [mm]			
Pre-operatively	23 (21–25)	22 (19–24)	0.572
Admission to ICU	15 (13–20)	15 (12–21)	0.809
24 h postoperatively	22 (20–25)	24 (20–28)	0.424
TRAP [AU]			
Pre-operatively	119 (82–159)	103 (93–143)	0.501
Admission to ICU	139 (93–159)	116 (85–149)	0.572
24 h postoperatively	147 (116–157)	134 (129–158)	0.647
ASPI [AU]			
Pre-operatively	20 (13–43)	10 (7–30)	0.183
Admission to ICU	20 (12–48)	24 (5–52)	0.893
24 h postoperatively	33 (19–48)	34 (23–41)	0.767
ADP [AU]			
Pre-operatively	64 (43–78)	63 (35–71)	0.434
Admission to ICU	61 (44–72)	45 (33–82)	0.476
24 h postoperatively	71 (53–85)	71 (58–90)	0.727

Results are given as median (IQR). Reference ranges of the local laboratory: Platelets 150–370/nl; aPTT 26–40s; thromboplastin time 70–130%; fibrinogen 1.6–4.0 g/l. Reference ranges for activated rotational thromboelastometry: CT (Intem) 137–246 s; CT (Extem) 42–74 s; MCF (Fibtem) 9-25 mm. Reference ranges for multiple aggregometry: TRAP 84–128 AU; ASPI 71–115 AU; ADP 57–113 AU. Differences were analysed using nonparametric Mann-Whitney U test for two independent samples with α = 0.05 (two-sided). Significant tests are marked with *

The secondary outcome parameters duration of mechanical ventilation postoperatively and the incidence of renal replacement therapy are also included in Table 1. Crystalloid/colloid infusions and urine output did not differ between the groups over the observation period (data not shown). Analyses were repeated after propensity matching. No significant differences regarding the impact of possible confounders were observed.

Protocol deviations occurred in three patients. The first case received 400 ml of FFP in the conventional group in the initial phase of this study. The other two protocol deviations were the transfusion of two units of platelets at once, one also in the initial phase of the study. The second occurred intraoperatively prior to chest closure due to diffuse bleeding.

## Discussion

A point-of-care guided transfusion algorithm did not result in less bleeding than a transfusion algorithm based on conventional coagulation test results in our study population. Transfusion requirements of RBCs and FFPs did not differ, while platelets were transfused in the PoC group only. There was no clinically significant difference in the course of coagulation parameters, duration of mechanical ventilation, or incidence of renal replacement therapy. Bleeding was less frequent and blood loss was lower than expected. Therefore, blood loss via chest tube drainage was not suitable to distinguish between a PoC- or central lab-guided transfusion algorithm. This may be attributed to the fact that surgery at high risk for perioperative bleeding may not sufficiently be defined only by the procedure but needs to include individual patient risk factors. In patients with insignificant bleeding point-of-care diagnostics do not seem to improve treatment and outcome as we detected no measurable difference. However, the low blood losses resulted in the early termination of this study.

Our results are in contrast to published studies showing a superiority of PoC guided algorithms in terms of reducing allogeneic blood transfusions [20, 23, 24]. Undoubtedly, transfusion algorithms - not covering RBCs - are recommended to be used in severely bleeding patients only, as non-bleeding patients usually do not need any therapeutic intervention [16]. The relatively low blood losses postoperatively that usually do not require substitution with blood products may also explain why the haemostatic algorithm using PoC technique we used for this study was not only not superior to an algorithm driven by conventional coagulation parameters, but may, in contrast, lead to a higher transfusion requirement when closely followed.

According to the EACTS/EACTA Guidelines on blood management, the first step towards creating algorithms is to identify patients at high risks for bleeding which are, following our results, not sufficiently defined by high-risk surgery only [33]. The anticipated chest tube drainage volume in our study which qualified patients for enrolment was unexpectedly low. We designed our study with the aim to compare patients undergoing surgery at high risk for bleeding without further risk factors for coagulopathy that might reduce comparability. This led to a sample of rather healthy subjects whose main risk for bleeding was the procedure itself. In this group, overall blood loss, incidence of relevant blood loss and transfusion requirements were remarkably below those of other studies [15, 21, 23, 24, 34]. This supports the idea that the definition of high-risk surgery should not only be made by the procedure itself but include individual patient risk factors, since better blood conservation technics have been established in the recent time [12, 35].

Nevertheless, there is a heterogeneity in the definition of bleeding. Our study enrolled patients preoperatively with a high risk for bleeding as predicted by the surgical procedure [3]. Bleeding was defined as intraoperative oozing delaying chest closure or as blood loss exceeding 1.5 ml/kgBW/hour in two consecutive hours or 4 ml/kgBW/hour for at least 30 min postoperatively. The study of Weber et al., however, enrolled patients with “diffuse bleeding from capillary beds at wound surfaces requiring haemostatic therapy as assessed by the anaesthesiologist and surgeon by inspecting the operative field and/or intraoperative or postoperative blood loss exceeding 250ml/h or 50ml/10min” [24]. 88% of their patients were enrolled intraoperatively without detailing which “coagulopathic” patients bled more than 250 ml/h or 50 ml/10 min or were bleeding diffusely. This inclusion criterion might be less precise compared to another method described, i.e. quantifying bleeding intraoperatively by packing with and weighing of sponges [36].

Furthermore, Weber et al. enrolled 100 of 152 eligible patients intraoperatively [24]. A larger sample of 1144 patients assessed according to the universal definition of perioperative bleeding found a distribution of 51.4% patients experiencing insignificant bleeding only, mild bleeding in 14.9% or moderate bleeding in 24% of patients, while severe or massive bleeding occurred in 9.8% of the patients [6].

Our study included 25 patients with a high risk of bleeding, of whom 20% experienced a blood loss requiring transfusion of haemostatic blood products excluding RBCs. The majority of patients suffered insignificant bleeding; blood losses were much lower than in previously published studies [15, 21, 23, 24, 34], that may explain the different results. There are several potential reasons for these differences: advances in surgical technique, implementation of patient blood management programs and even more important a more meticulous haemostasis by cardiac surgeons who were not blinded to the study group allocation.

For the secondary outcome parameter, transfusion requirements, our results are also contrary to earlier work [20]. This might be due to the lower incidence of bleeding in general. However, despite the small sample, the incidence of platelet transfusions in the PoC group might require more attention, as 36% of the patients were transfused compared to none in the conventional group. The decision to transfuse platelet concentrates should not be based on lab testing alone, but also on the clinical condition of the patient and the amount of blood loss. This aspect should especially be considered when facing patients that are rated as at high risk for bleeding, therefore being examined with PoC parameters but finally not having significant blood losses. Future clinical studies addressing PoC-based transfusion triggers for platelets in bleeding patients might help shedding light on this issue.

This study has some limitations: First, the limited number of included patients results in lack of power [37]. Second, we recognized three protocol deviations accounting for 12% of patients. This may have caused some bias influencing the results [38]. However, 88% were treated according to the treatment protocol, which is a comparably high rate of protocol adherence. Third, blinding was not possible in this study as PoC coagulation testing started in the operation theatre, which may have influenced surgeons to perform a better surgical haemostasis in all patients. Furthermore, due to the hospital information system, blinding of the attending physicians to the conventional coagulation parameters was not possible, although the transfusion protocol did not allow for transfusions based on those parameters in the PoC group. Fourth, criteria to exclude patients with haemostasis defects were strict, causing a low overall percentage of eligibility. This might reduce the generalizability of this study. Last, our definition of bleeding risk by surgical procedure did not include all patients with a high risk of bleeding, e.g. patients on dual antiplatelet therapy, in whom platelet function testing may have yielded better results than a platelet count driven transfusion algorithm. However, the type of surgery remains an important factor to define the risk of bleeding in cardiac surgery [21, 24, 25].

## Conclusion

This study suggests that blood losses and transfusion amounts did not differ comparing PoC- and central lab-driven transfusion algorithms in high-risk cardiac surgery, when drainage losses are small (< 500 ml/12 h) and surgeons not blinded to study group allocation. The definition of high-risk for bleeding should possibly include individual risk factors rather than procedural factors only. Physicians should take these limitations into account when considering PoC measurements for this patient group. These results need re-evaluation in a larger prospective and randomized multicentre trial implementing explicit and restrictive goal-directed transfusion algorithms in both groups.

## Appendix

Exclusion Criteria:

- ▪ age < 18 or > 80 years
- ▪ known haemophilia
- ▪ known thrombophilia
- ▪ known hereditary thrombocytopathy
- ▪ hereditary or acquired coagulation disorder (not including single antiplatelet therapy)
- ▪ active endocarditis
- ▪ ejection fraction < 30%
- ▪ BSA < 1.8 sqm
- ▪ planned aortic arch surgery
- ▪ preoperative thrombocytopenia < 150/nl
- ▪ underlying haemostaseological disease
- ▪ preoperative anaemia
- ▪ liver cirrhosis Child B or higher
- ▪ preoperative creatinine > 2 mg/dl
- ▪ terminal renal insufficiency requiring dialysis
- ▪ vitamin k antagonists during 5 days prior to surgery
- ▪ pregnant or breastfeeding women
- ▪ known allergy against allogeneic blood products or coagulation factors
- ▪ refusal of blood transfusions
- ▪ any concomitant investigational agent or participation in another trial

## Abbreviations

ACT: Activated clotting time
aPTT: Activated Partial Thromboplastin Time
BIS: Bispectral index
CABG: Coronary artery bypass graft
CPB: Cardio-pulmonary bypass
FFP: Fresh frozen plasma
IQR: Interquartile ranges
MEA: Multiple electrode aggregometry
PoC: Point-of-care
RBC: Packed red blood cells
ROTEM: Rotational thromboelastometry
TRALI: Transfusion associated lung injury
